# Child poverty in armed conflict regions of Africa: A scoping review protocol

**DOI:** 10.1371/journal.pone.0352651

**Published:** 2026-06-29

**Authors:** Jasper Damaris Raja, Solina Richter, Kimberly Jarvis

**Affiliations:** 1 College of Nursing, University of Saskatchewan, Saskatoon, Saskatchewan, Canada; 2 Department of Health Studies, University of South Africa, Pretoria, South Africa; 3 Faculty of Nursing, Memorial University of Newfoundland, St. John’s, Newfoundland and Labrador, Canada; World Health Organization, CONGO

## Abstract

**Objective:**

In this scoping review, we aim to identify and map the dimensions and underrepresented multidimensional aspects of child poverty in armed conflict regions of Africa. We will develop a framework for the multidimensional aspects of child poverty in conflict-affected countries in Africa to address poverty as per United Nations (UN) Sustainable Development Goal (SDG) 1. This review will provide a comprehensive understanding of how the multidimensional aspects of child poverty are unique in conflict regions, adding layers to the existing Global Multidimensional Poverty Index (MPI) and United Nations Children’s Fund’s (UNICEF) Multiple Overlapping Deprivation Analysis (MODA).

**Introduction:**

Armed conflict affects many countries globally, with Africa ranking second in conflict prevalence. One in four African children experience significant deprivation. No existing scoping or systematic review has examined the multidimensional nature of child poverty in African conflict settings.

**Inclusion criteria:**

This review will include studies involving children from birth to 18 years of age living in African armed conflict contexts and will include at least one dimension of child poverty.

**Methods:**

This Priori protocol will follow the Joanna Briggs Institute (JBI) methodology to recognize the methodologically rigorous approaches of conducting scoping reviews. This approach will be used to plan, standardize, and increase the transparency of the scoping review outline. Eligible sources will include peer-reviewed publications in English, French, or Portuguese from Medline, Embase, CINAHL, Scopus, and WHO Africa Index Medicus (AIM), as well as grey literature from nongovernmental and international agencies, ProQuest, and the Open Access Theses and Dissertations (OATD) database. This review will use a comprehensive search strategy, manage screening and data extraction through Covidence integrated with Zotero, and use Google Translate for preliminary translation of French and Portuguese texts. This protocol, registered with Open Science Framework (OSF), outlines a rigorous and transparent approach to mapping existing evidence on child poverty in African armed conflict settings.

## Introduction

Worldwide, armed conflicts have increased drastically after World War II, reshaping global patterns of conflict over the past 75 years [1]. Armed conflict is defined as a dispute or civil disorder that involves the use of force, resulting in at least 25 battle-related deaths per year [2]. Armed conflict threatens peace and development, weakens economy, health, and infrastructure, and endangers civilian lives [3,4]. Approximately 246 million children live in conflict-lethal war environments worldwide [5,6]. Africa ranks second among world regions in the number of active conflicts, with more than 35 Non-International Armed Conflicts (NIACs) occurring across the continent [7]. Such conflicts affect one in every four children in Africa, contributing to rising levels of poverty [8]. Child poverty affects children at twice the rate of adults and is particularly higher in fragile (38.8%) than in non-fragile conflict states (10.1%), where overlapping risks of multidimensional deprivation exist [9–13].

Although child poverty is traditionally viewed through household economy monetary indicators, these indicators do not capture the multiple deprivations experienced by children in conflict settings who earn less than US $3 per day [14–16]. Researchers increasingly recognize the multiple deprivations and capture diverse aspects of child poverty [13,14,17,18]. Child poverty is multifaceted, intricate, and inclusive of a lack of resources from tangible and non-tangible multidimensions such as health, education, and living standards, inclusive of nutrition, child mortality, years of schooling, school attendance, water availability, housing, information, electricity, assets, cooking fuel, and sanitation, mounted on the needs and rights of children [14,17–19]. Literature suggests that multidimensional child poverty is widespread in sub-Saharan Africa armed conflict, affecting 6 in 10 children [11,13,20–24].

The Global MPI assesses child poverty across health, education, and living standards, yet conflict intensifies these deprivations [17,18,21]. Proximity to conflict increases infant mortality fivefold and contributes to wasting, stunting, and undernutrition [25–29]. The education dimension is also heavily affected. Conflict limits children’s learning opportunities and reduces the quality of schooling, leading to lower attendance, wider gender gaps, and poorer academic performance [30–35]. The living standards dimension declines as conflict disrupts access to basic needs. Indicators such as cooking fuel, improved sanitation, safe drinking water, electricity, adequate housing, and essential household assets all deteriorate in conflict settings [17,36–39].

However, neither the Global MPI nor the UNICEF’s MODA fully captures the conflict specific dimensions of child poverty [14,17,40]. Emerging evidence highlights additional deprivation domains, such as safety and security, legal status, child protection, psychosocial well-being, mobility restrictions, lack of sports activity, psychological and emotional distress, which particularly in conflict-affected settings have significant effects on children [40]. Recent scholarship emphasizes the need for a conflict-specific child poverty framework. A framework guided by the integrating of Global MPI, UNICEF’s MODA, and conflict specific dimensions is therefore essential for understanding child poverty in African conflict zones. The multidimensional aspects (at least one or more) that overlap, affect children’s health, and contribute to multidimensional poverty in armed conflict regions of Africa [19,41]. Multidimensional poverty is detrimental, creating endless challenges and trapping children in multiple crises, leaving the SDG 1 of ending poverty unresolved.

Although there is a growing body of research on specific forms of deprivation such as stunting, wasting, undernutrition, education, and access to safe drinking water, there is still limited literature on child poverty as a multidimensional issue, especially within conflict-affected African countries [17,25–29,36–39].

Existing literature reviews focus on single dimensions of child poverty, such as undernutrition, leaving multidimensional patterns unexplored [27]. A preliminary search of MEDLINE, the Cochrane Database of Systematic Reviews, and JBI Evidence Synthesis confirmed the absence of a comprehensive scoping review on this topic. Guided by the JBI methodology for scoping reviews this review aims to systematically identify and map the multidimensional literature on child poverty conflict settings and identify underrepresented dimensions. This would guide future nursing research and practice to alleviate poverty in armed conflict regions of Africa [42].

### Review question

What is known from the existing literature about multidimensional poverty among children aged birth to 18 years living in areas of Africa affected by armed conflict?

### Objectives

The objective is to identify:

1. the components of child poverty in armed conflict affected countries in Africa
2. the underrepresented multidimensional aspects of child poverty in the literature on armed conflict affected countries in Africa.

## Methods

The proposed scoping review will be conducted using the JBI methodology for scoping reviews [43]. The protocol is registered with OSF (https://doi.org/10.17605/OSF.IO/EYGP2). Any deviations from the protocol will be reported and justified in the review manuscript.

### Study design

This scoping review is a type of evidence synthesis that aims to identify and map the breadth of the existing and emerging evidence, clarify concepts, and identify research gaps available on child poverty in armed conflict regions of Africa [44].

### Inclusion and exclusion criteria

The following is an exclusive list of inclusion criteria. The authors will exclude other articles that do not fulfill the inclusion criteria**.**

Population: This scoping review will examine articles that include children as aged from birth to 18 years who live in areas of Africa affected by armed conflict [45]. This review will include articles that are all-inclusive, encompassing all children, regardless of their gender or other demographic characteristics, with age as the only restriction, including mixed- age populations if child- specific findings are reported in the literature.

Concept: Poverty, a multidimensional phenomenon is under examination in this scoping review of regions of armed conflict. For this review, child poverty is defined as the state of children who lack tangible and intangible living resources, including all existing and missing dimensions of the Global MPI, and UNICEF’s MODA and reflecting multistage conflict dimensions. The existing dimensions are health (nutrition, child mortality), education (year of schooling and school attendance), living standards, inclusive of indicators (cooking fuel, sanitation, water, electricity, housing, assets) information, clothing, environmental, economic, cultural, relational, opportunity, and social resources [17–21]. There is overlap between conflict-sensitive dimensions and Global MPI in some areas, such as housing, water, nutrition, and sanitation [40].

The other standalone dimensions related to conflict are information, legal status (documentation -child is missing documents like birth certificate, unaccompanied and separated), child protection (eviction risks, child marriage, child labour), safety (children and family do not feel safe (zero-5 years), unsafe to go to washing and toilet facilities for children (6–17 years), food security (smaller meals, fewer meals), psychological and emotional distress [40]. During the screening stage of this scoping review, studies that address any dimension of child poverty will be included to meet Objective 1. For Objective 2, full-text articles will be selected if they highlight one or more dimensions of child poverty and help illustrate its multidimensional nature. This means the review will include studies that focus on any single dimension of child poverty, as well as those that examine multiple dimensions.

Armed Conflict: Research articles that reflect child poverty in African countries affected by armed conflict will be considered. For this scoping review, armed conflict is defined as a dispute or civil disorder that involves the use of force, resulting in at least 25 battle-related deaths per year [2]. The review encompasses all types of conflicts, including interstate, intrastate, and intergroup conflicts, relying on the author’s descriptions. The keywords, title, and abstract for all relevant concepts (children, armed conflict, and Africa) are in S1 File.

Context: This review will consider research papers stating that the study setting is in Africa. Africa is a continent comprising 54 countries and six island nations [46].

### Types and sources of evidence

Quantitative, qualitative, mixed-methods studies, dissertations, theses, will be included. The articles will include contributions from both community and hospital settings. To comprehensively address child poverty, the review will encompass all relevant literature, with no restrictions on publication years. The review will include research resources published in English, French, and Portuguese as the dominant communication languages compared to other (non-colonized) African languages.

Source selection is based entirely on the protocol’s inclusion criteria from Medline, Embase, CINAHL, Scopus, and WHO, as well as AIM. Reports from nongovernmental and international websites that are currently working with children living in poverty in armed conflict regions of Africa, including the WHO, UNICEF, UNFPA, and UNDP will be included. Theses and dissertations from ProQuest and OATD will be included. After the search, citations were uploaded to Zotero as a Research Information System (RIS) file to remove duplicates. The review utilizes Covidence, an online software tool for producing literature reviews through citation screening, full-text review, extraction of study characteristics and outcomes, and export of data and references. Titles and abstracts will be reviewed against the inclusion criteria by two independent reviewers as per JBI methodology. The review will include relevant articles examined through a full-text analysis by two independent reviewers. If there is any exclusion, the pertinent reason and all details will be captured in the final manuscript. Two authors started screening and piloting a subset of 50 potentially relevant articles to ensure consistency. Any disagreement will be resolved by a third reviewer through discussion. The PRISMA-ScR extension for scoping reviews (Preferred Reporting Items for Systematic and Meta-Analyses for Scoping Reviews) flow diagram will graphically depict the movement of sources through the search results in the final manuscript, including studies from both databases and other methods, such as hand searches of documents and reports, as mentioned in S1 Fig. [47]: additionally we provide a PRISMA-P 2015 checklist [48] as supporting information (S1 Table). This review will include a flow chart containing 20 final reporting items and two optional items, allowing the authors to provide the title, structured summary, rationale, and example of good reporting for each item. In developing a deeper understanding of terminology, fundamental concepts and key items to report for scoping reviews will be included in the final manuscript. Considering the broad nature of the search and review, the authors will incorporate new, relevant information into the final manuscript. Deidentified research findings will be made available upon completion and publication of the study. The record screening and data extraction will be completed in 2026, and results will be expected in 2027, given the scope, volume, and complexity of the review, which interlocks with other parts of this research project.

### Search strategy

The search strategy will aim to locate both published and unpublished studies. The search will locate a variety of subjects based in the medical and social sciences fields, such as Medline, Embase, CINAHL, Scopus, and WHO (AIM) and grey literature from nongovernmental and international websites that are currently working with children living in poverty in armed conflict regions of Africa, WHO, UNICEF, UNFPA, and UNDP. Theses and dissertations from ProQuest and OATD will be included. Based on the review question, a list of keywords and search terms from the literature were identified. An initial search based on preliminary search results guided the selection of keywords and indexing terms.

A preliminary search was conducted in Medline, tracing articles. The process was rigorous to understand the complex concept and identify gaps in the evidence with the consultation of a librarian. Seed articles, encompassing all three concepts (armed conflict, children, and Africa) during the preliminary search were identified. Ninety seed articles from the preliminary search matched the advanced search, strengthening and confirming the rigour of the search.

To compile a comprehensive list of databases for mapping articles from Africa’s low-income countries and conflict regions, a detailed list of databases, search platforms, relevant links, and websites was identified, including those that search grey literature sources. Initial searches across various databases were recorded and shared with the team as a search history or a screenshot for discussion, decision-making, and transparency. This step helped narrow down the databases with the librarian. The search draft is partial, incomplete, and requires articles from other databases. The review will include a future hand search of WHO, UNDP, UNICEF, and UNFPA documentation and nongovernmental reports.

This protocol includes a search strategy from Medline (S1 File), to improve clarity and transparency. Then we translated the search from Medline to other databases using a polyglot accelerator tool designed for search string translation in research [49]. The review will include studies published in English, French, and Portuguese. The authors are open to a wide range of data to avoid restrictions on the concept of armed conflict.

### Data extraction

Two independent reviewers will extract data from the journal articles included in the scoping review using a data extraction tool developed by the reviewers, as outlined in the final manuscript. The draft data extraction tool will be adjusted and refined as necessary during the extraction, as it is an iterative process (S2 Table). The extracted data will include specific details about the age subgroup of participants (children), incorporating gender and child -specific information such as street involvement, being an orphan, HIV status, disability, unaccompanied minors, peacekeeper fathered, children conceived by rape (rape babies), children born of war (CBOW), and military children. The data extraction on the armed conflict concept will incorporate the author’s descriptions in the literature, such as the type of conflict (state-based conflict, non-state conflict, one-sided violence defined by UDCP), duration, and conflict stage (peri, during, and post-conflict). Finally, for the African concept, the information on the nation of Africa and the year of publication will be included. Additionally, if the scoping review includes dimensions of poverty, either one or more relevant to the review question and objectives, they will be mapped to the multidimensional aspects of child poverty. At the beginning of data extraction, the authors will conduct a pilot test to analyse the first 10 papers, ensure consistency and clarity, and provide a detailed description for the reader. The review will include a final, completed tool that the authors will present in the scoping review report after independently cross-checking the extracted data. The third reviewer will resolve any disagreements between authors through discussion. Authors of the article will be contacted to request additional information, when required.

### Data analysis and presentation

Considering the complexity of the topic, the multidimensional aspects of child poverty within the conflict zones framework will be developed. The existing global MPI**,** UNICEF’s MODA, and conflict-caused emergency context dimensions will be mapped along with all emerging dimensions [40]. The reviewers will extract all the descriptive information about the source: authors, year of publication, country of origin, region of Africa, aims or purpose, age specific, gender specific, child- specific, dimensions of child poverty either one or more, types, duration, and stages of conflict such as peri, during, and post conflict as per author’s description and methodology, and method. Qualitative content analysis will aid in mapping the multidimensional concepts of child poverty. The reviewer will use a Microsoft Excel sheet to extract the data. A draft data extraction tool is attached as a supporting document (S2 Table). The third reviewer will perform random checks on the extracted information. Only relevant extracted information to review research questions will be reported. As the extraction is an iterative process, any deviation will be reported. The reviewer will pilot the extraction tool to identify missing, redundant, and unclear information in each data source.

The authors will extract data for graphic display in tabular form, providing a descriptive summary that aligns with the objectives of this scoping review and the PCC framework (population, concept, and context) as a benchmark for the review question and objectives. The tabulated results will provide core knowledge on the multidimensional aspects of child poverty in African armed conflict regions. The review will provide a synthesis of existing data to identify gaps in the literature and guide future research. As some parts of the scoping review are iterative, the authors will report any deviations from the protocol in the review manuscript to maintain transparency. No datasets were generated or analysed during this current study. The extracted data and data sources will be available for the public in the final manuscript upon study completion. Any additional data will be available on request from the first author.

### Ethics and dissemination

Ethical approval is not required to conduct this scoping review. Only published articles, reports, and websites will be used. Findings from this study will be presented to the general public, at scientific conferences, and published in a peer- reviewed journal.

## Supporting information

S1 File Search strategy in Medline. Full search strings including keywords, MeSH terms, and Boolean operators, used to search OVID Medline. The search was performed on November 1, 2025.
(DOCX)

S1 Fig Adapted PRISMA-ScR 2020. PRISMA flowchart produced by Covidence reports the identification, screening, and inclusion of studies via databases and other methods.
(TIF)

S1 Table PRISMA-P 2015 Checklist. This checklist has been adapted for use with protocol submissions to *Systematic Reviews* from Table 3 in Moher D et al: Preferred reporting items for systematic review and meta-analysis protocols (PRISMA-P) 2015 statement. *Systematic Reviews* 2015 4:1.
(DOCX)

S2 Table Draft data extraction tool. This draft table includes detailed descriptions of study information, characteristics, population, children, armed conflict, child poverty, results & outcomes, gaps and relevance.
(DOCX)
