# Supplementary material for: Child poverty in armed conflict regions of Africa: A scoping review protocol
Source: PLoS One. 2026 Jun 29;21(6):e0352651. doi: 10.1371/journal.pone.0352651 (PMC13313329; doi:10.1371/journal.pone.0352651)
Supplement: S1 File — Full search strings including keywords, MeSH terms, and Boolean operators, used to search OVID Medline. The search was performed on November 1, 2025. (DOCX) [file pone.0352651.s001.docx]

**S1 File**

**Searches Conducted on Ovid MEDLINE (1^st^ November 2025).**

1"Warfare and armed conflicts"/ or armed conflicts/ or warfare/ or war crimes/ or ethnic cleansing/ or genocide/

26690

2 (warfare or war or wars or armed conflict* or terroris* or persistent conflict* or prolonged conflict* or political conflict* or persistent violence* or prolonged violence* or political violence* or armed violence* or humanitarian emergenc* or complex emergenc*).ti,ab,kf. 76456

3 ((conflict or conflicts or fragil* or vulnerab*) adj3 (area or areas or zone or zones or setting or settings or situations or states)).ti,ab,kf. 10557

4 (Civil disorder* or riot* or civil unrest).ti,ab,kf.

1157

5 (ethnic cleansing or ethnic conflict* or genocide).ti,ab,kf.

1041

6 1 or 2 or 3 or 4 or 5 [Armed Conflict]

97907

7 exp child/ or exp infant/ or adolescent/ or exp pediatrics/ or child, abandoned/ or exp child, exceptional/ or child, orphaned/ or child, unwanted/ or minors/ or (pediatric* or paediatric* or (child* not childbearing) or newborn* or congenital* or infan* or baby or babies or neonat* or pre-term or preterm* or premature birth* or NICU or preschool* or pre-school* or kindergarten* or kindergarden* or elementary school* or nursery school* or (day care* not adult*) or schoolchild* or toddler* or boy or boys or girl* or middle school* or pubescen* or juvenile* or teen* or youth* or high school* or adolesc* or pre-pubesc* or prepubesc*).mp. or (child* or adolesc* or pediat* or paediat*).jn.

5552429

8 Under five*.ti,ab,kf.

10416

9 7 or 8 [Children] 554107

10 exp Africa/ or (Africa* or Algeria or Angola or Benin or Botswana or "Burkina Faso" or Burundi or "Cabo Verde" or "Cape Verde" or Cameroon or "Central African Republic" or Chad or Comoros or Congo or "Cote d'Ivoire" or "Ivory Coast" or Djibouti or Egypt or "Equatorial Guinea" or Eritrea or Eswatini or Ethiopia or Gabon or Gambia or Ghana or Guinea or "Guinea-Bissau" or Kenya or Lesotho or Liberia or Libya or Madagascar or Malawi or Mali or Mauritania or Mauritius or Morocco or Mozambique or Namibia or Niger or Nigeria or Rwanda or "Sao Tome" or Principe or Senegal or Seychelles or "Sierra Leone" or Somalia or "South Africa" or Sudan or Tanzania or Togo or Tunisia or Uganda or Zambia or Zimbabwe).mp.

872350

11 exp africa, northern/ or (sudan* or western sahara* or algeria* or egypt* or libya* or morocc* or tunisia* or Cairo or Rabat or Casablanca or Tripoli or Algiers or Fes or Marrakesh or Tunis or Carthage or (Alexandria not (VA or Virginia)) or Tangier or Kairouan or Essaouira or Luxor or Bizerte or "El Aaiun" or Sousse or Oran or Annaba or Constantine or Biskra or Chefchaoouen or Skikda or "Sharm El Sheikh" or Volubilis or "El Oued" or Meknes or Hippo Regius or Djemila or Sfax or Tataouine or port Said or "Ait Benhaddou" or Benghazi or Juba or Tamanrasette or merzouga or "El Djem" or oujda or Matmata or Ghat or Tabessa or Giza or Marj or Ifrane or "M'Hamid El Ghizlane" or Agadir or Tetouan or "Shubra El Kheima" or Tobruk or Khartoum or Nyala or Kassala or Ubayyid or Kosti or Wad Madani or Qadarif or Al-Fashir or Daein or Damazin or Geneina or Merowe or (north* adj2 africa*)).ti,ab,kf. 108666

12 exp Africa, Eastern/ or (east* adj2 africa*).mp. or British Indian Ocean Territory.mp. or Burundi*.mp. or Comoros.mp. or Djibouti*.mp. or Eritrea*.mp. or Ethiopia*.mp. or Kenya*.mp. or Madagascar.mp. or Malawi.mp. or Mauritius.mp. or Mayotte.mp. or Mozambique.mp. or Reunion.mp. or Rwanda*.mp. or Seychelles.mp. or Somalia*.mp. or Sudan*.mp. or Tanzania*.mp. or Uganda*.mp. or Zambia.mp. or Zimbabwe.mp. or Crozet Islands.mp. or Iles Crozet.mp. or Scattered Islands.mp. or Iles Eparses.mp. or Addis Ababa.mp. or Asmara.mp. or Anananarivo.mp. or Arusha.mp. or Axum.mp. or Bahir Dar.mp. or Berbera.mp. or Bulawayo.mp. or Dese.mp. or Eldoret.mp. or Garissa.mp. or Geita.mp. or Gondar.mp. or Great Rift Valley.mp. or Hargeisa.mp. or Hargeysa.mp. or Hola.mp. or Jinja.mp. or Iringa.mp. or Kigoma.mp. or Jimma.mp. or Korogwe.mp. or Nairobi.mp. or "Dar es Salaam".mp. or Mombasa.mp. or Mogadishu.mp. or Dodomoa.mp. or Bujumbura.mp. or Mbeya.mp. or Lusaka.mp. or Harare.mp. or Kakamega.mp. or Kampala.mp. or Kigali.mp. or Kire Dawa.mp. or Kikuyu.mp. or Kisumu.mp. or Kitale.mp. or Kitui.mp. or Lilongwe.mp. or Lake Victoria.mp. or Lake Tanganyika.mp. or Lamu.mp. or Lodwar.mp. or Lokichogio.mp. or Malindi.mp. or Machakos.mp. or Marka.mp. or Machakos.mp. or Maputo.mp. or Maralal.mp. or Mek'ele.mp. or Meru.mp. or Musoma.mp. or Mtwara.mp. or Mumias.mp. or Moshi.mp. or Moroni.mp. or Morogoro.mp. or Mwanza.mp. or Naivasha.mp. or Nanyuki.mp. or Nakuru.mp. or Namanga.mp. or Nyeri.mp. or Port Louis.mp. or Puntland*.mp. or Nyahururu.mp. or Kismayo.mp. or Ruiru.mp. or Rwenzori Mountains.mp. or Sinyanga.mp. or Songea.mp. or tanga.mp. or Tabora.mp. or voi.mp. or webuye.mp. or Zanzibar.mp. or ((Adiharush or Ali-Addeh or Alinjugur or Buramino or Dadaab or Dagahaley or Dollo Ado or Fugnido or Hagadera or Hilaweyn or Ifo or Kakuma or Kambioos or Kayaka II or Kobe or Kyangwali or Nakivale or Nyarugusu or Wad Sherife or Bokolmanyo or Melkadida or Rwamanja) adj5 (camp or refug*)).ti,ab,kf.

193685

13 exp africa, central/ or ((africa adj2 central) or angola or cameroon* or chad or tchad or congo* or DRC or equatorial guinea* or gabon* or Sao Tome or Principe or Luanda or lobito or kuito or huambo or Malanje or Douala or Yaounde or Bamenda or Garoua of Bafoussam or Nganoundere or Maroua or Kouosseri or Buena or Kumba or "N'Djamena" or Moundou or Bangui or Bimbo or Brazzaville or Point Noire or Kinshasa or Lubumbashi or Leopoldville or Elizabethville or Mbuji Mayi or Bakwanga or Bukavu or Costermansville or Kananga or Luluabourg or Kisangani or Stanleyville or Tshikapa or Koalwezi or Likasi or Jadotville or Goma or Kikwit or Uvira or Bunia or Mbandaka or Coquilhatville or Matadi or Butembo or Kabinda or Mwene Ditu or Isiro or Paulis or Boma or Kindu or Bata or Malabo or Libreville).ti,ab,kf.

54732

14 exp africa, western/ or ((africa* adj2 west*) or benin* or burkina fas* or cape verd* or cabo verd* or ivory coast or "cote d'ivoire*" or gambia* or ghana* or guinea* or bissau or liberia* or (mali not fowl) or malian or mauritania* or nigeria* or senegal* or sierra leon* or togo*).mp. or (Lagos or Accra or Abidjan or Dakar or Abobo or Abuja or Freetown or Ouagadougou or Conakry or Lome or Bamako or Cotonou or Kumasi or Monrovia or Ibadan or Kano or Port Harcourt or Benin City or Porto Novo or Niamey or Yamoussoukro or Banjul or Timbuktu or Djenne or Abomeyu or Zaria or Tamale or Jos or Cape Coast or Maidugul or Aba or Gao or Calabar or Warri or Maiduguri or Bobo Dioulasso or Parakou or Djougou or Bohicon or Sekondi Takoradi or Sunyani or Obuasi or Teshie or Tema or Sikasso or Kalabankoro or Nouakchott or Dakhlet Nouadhibou or Benin City or Port Harcourt or Ilorin or Kaduna or Enugu or Ikorodu or Onitsha or Bauchi or Akure or Abeokuta or Sokoto or Bouake or Makeni or Kaduan or Sosgbo or Osogbo or Gombe or Ilesa or Badagry or makurdi or Sagamu or Iseyin or obbomosho or Awka or Ado Ekiti or Nsukka or Ikeja or Katsina or Okene or Lafia or Minna or Ondo city or Umuahia or Calabar or Yola or Pikine or Touba or "Thies Nones" or Saint Louis or Kolak or Ziguinch or (San Pedro not (Spain or Mexico or Argentina or California or United States or Italy)) or Bandama or Daloa or Owerri or Kandi or Ifi or Dakar or Ogbomosho or Divo or Korhogo).ti,ab,kf.

330647

15 exp africa, southern/ or ((africa* adj2 south*) or angola* or botswana* or lesotho* or malawi* or mozambiq* or namibia* or swaziland or zambia* or zimbabwe or Zulu or Tsonga or Xhosa or Swazi or Ndebele or Tswana or Sotho or Shona people or BaLunda or Mbundu or Ovimbundu or Chaga or Sukuma or Pretoria or Cape Town or Johannesburg or Durban or Port Elizabeth or Bloemfontein or Windhoek or Maseru or Pietermaritz or (Kimberley not Australia) or Nespruit or Soweto or Polokwane or Limpopo or Rustenburg or Mahikeng or Oudtshroom or Stellenbosch or Paarl or Gaborone or Luanda or Cabinda or Huambo or Lubango or Kuit or Malanje or Lobito or Lilongwe or Blantyre or Mzuzu or Maputo or Matola or Beira or Nampula or Chimoio or Nacala or Quelimane or Lusaka or Kitwe or Ndola or Kabwe or Copperbelt or Harare or Bulawayo or Chitungwiza or Mutare or Masvingo or Monashonaland or Manicaland).ti,ab,kf.

135693

16 10 or 11 or 12 or 13 or 14 or 15 [Africa]

951347

17 6 and 9 and 16 [All Three Concepts]

2727
