# Supplementary material for: Child poverty in armed conflict regions of Africa: A scoping review protocol
Source: PLoS One. 2026 Jun 29;21(6):e0352651. doi: 10.1371/journal.pone.0352651 (PMC13313329; doi:10.1371/journal.pone.0352651)
Supplement: S2 Table — This draft table includes detailed descriptions of study information, characteristics, population, children, armed conflict, child poverty, results & outcomes, gaps and relevance. (DOCX) [file pone.0352651.s004.docx]

**S2 Table.** Draft data extraction instrument

| **Key domain** | **Data point** | **Description** |
| --- | --- | --- |
| Study identification | Title | Indicates the study title |
|  | Authors | Indicates the names of the authors (first and last name) |
|  | Year of publication | Indicates the year of publication |
|  | Country/ region/ island | Indicates the country of study, region, or Island (geographical location) |
|  | Source | This indicates database, journal or organization where the study as found |
| Study characteristics | Study type | Type of study (e.g., quantitative, qualitative, mixed methods or any other) |
|  | Methodology | If Qualitative (Grounded theory, Phenomenology, Ethnography, and Narrative inquiry). |
|  | Type of data | Primary or secondary |
|  | Objective | Primary aim or objective or review question of the study |
|  | SDG 1 | Any linkage to the SDG 1 |
|  | Setting of the study | Description of the study- hospital, community (rural, urban) school, or any other settings to specify |
|  | Other demographics | To include the specifications  Eg: Abduction from school |
|  | Sample size | Number of samples |
|  | Methods of data collection used | Survey, interviews, focused group, decolonizing methods, talking circles and observation. |
| Population | Age specific details | Newborn (0-28 days), infant (1 month to one year), toddler (1-3 year), preschooler (3-6 year), school age (6-12 year), adolescent (13-18 years),  Mixed age groups (found in literature) or  Life cycle stages (MODA)- 0-23 months, 24-59 months, 5-14 years, 15-17 years, any other specify |
|  | Gender specific details | Boy, girl, others specify |
| Children | Type of children | Children with parents, children living alone, children headed household, children with grandparents and extended families -aunts, uncles, cousins, street children, orphaned children, children with HIV, children with disabilities, unaccompanied children, peacekeeper fathered or blue helmet babies (children born to local women or girls after sexual relationships with UN peacekeeping personnel particularly in conflict region), children conceived as result of rape (rape babies), children born of war (CBOW: children fathers by foreign soldiers, peacekeepers, enemy combatants and born to local mothers during or after conflict) Military children are children dependents of personnel serving in the active, reserve or national guard forces, refugee children (individuals under 18 years old who have been forced to flee their home due to fear of persecution, conflict or violence). Others- specify. |
| Armed Conflict | Authors' words used to describe the conflict | For e.g: war, armed conflict, genocide |
|  | Secondary Data linked to ACLED* UCDP** |  |
|  | Type of armed conflict | State, non-state, between groups |
|  | Stage of Conflict | Pre, during, post-conflict |
| Child Poverty | Definition of child Poverty |  |
|  | Type of poverty | Absolute (severe poverty where income is below a minimum level required to meet basic needs) or relative (when a household receives 50 % less than average income, making them unable to meet the average standard of living in their society). |
|  | Unidimensional | Economics |
|  | Multidimensional child poverty  (Inclusive of Global MPI, MODA, and conflict sensitive emergency dimensions). | The dimensions include various areas such as:  **Health**  *Nutrition (*Undernutrition, Malnutrition,  Wasting, Stunting, Vaccination, Chronic Condition / Illness -For Example: Hypertension, Diabetes, Cancer & Others  *Child Mortality:* Specify (Neonatal/under five mortality)  **Education**  Year of schooling & school attendance  **Living Standards**  Availability of cooking fuel, sanitation,  Drinking water, electricity, housing, assets  **Legal Status** (documentation, Unaccompanied/separated), child protection (Eviction Risks, child marriage, child labour), **Safety (**Family does not feel safe for children (Zero-5 years, unsafe to go to washing and toilet facilities from children (6-17 years), **Food security-** smaller meals, fewer meals, psychological distress (mental), emotional distress.  information, political (right to speak),  opportunity (unequal, limited access to resources, experiences, and environments for healthy development, education, future success), social (refers to the lack of resources, social inclusion, and opportunities necessary for healthy development), environment,  clothing.  **Cultural** (resilience, kinship, communal sharing) |
|  | Multidimensional Poverty (more than 1) | More than one dimension for e.g: health & water, health & education,  Health & housing, water & sanitation (WASH) etc. |
| Results and Outcomes | Key findings | Major findings relevant to the study objectives |
|  | Conflict dimensions of child poverty | At various stages of conflict |
|  | Conflict multidimensional child poverty | At various stages of conflict |
| Gaps and relevance | Relevance to review objectives | Description of multidimensional poverty in African armed conflict areas |
|  | Identified gaps | Gaps or unanswered questions highlighted by the study |
