# Supplementary material for: Child poverty in armed conflict regions of Africa: A scoping review protocol
Source: PLoS One. 2026 Jun 29;21(6):e0352651. doi: 10.1371/journal.pone.0352651 (PMC13313329; doi:10.1371/journal.pone.0352651)
Supplement: Attachment — Submitted filename: Response to Reviewers.docx [file pone.0352651.s006.docx]

Jasper Damaris Raja

107 Cumberland Avenue S

 Wollaston Hall, Saskatoon.

Saskatchewan, Canada SK S7N2R6

+13068801877

[pxw184@mail.usask.ca](mailto:pxw184@mail.usask.ca)

April 4, 2026

Dear Editor- in -Chief,

We received the reviewed manuscript titled “***Child Poverty in Armed Conflict Regions of Africa: A Scoping Review Protocol***” from PLOS One. On behalf of myself and the other authors (Dr. Solina Richter & Dr. Kimberly Jarvis), I am **resubmitting the manuscript.** We have addressed **all points** raised by the **Academic Editor and the Reviewer.** Our responses are stated below with reference to the manuscript:

**Reviewer #1: Overall Assessment**
This protocol addresses an important and underexamined area at the intersection of armed conflict, multidimensional poverty, and child health in Africa. The topic is timely, policy-relevant, and appropriate for the journal. The use of JBI methodology, OSF registration and dual screening procedures are clear strength.
However, the protocol requires clarification in several core conceptual and methodological areas. The main concerns relate to, conceptual coherence around multidimensional poverty, consistency in inclusion criteria, operationalization of armed conflict exposure, and greater specificity regarding data extraction and synthesis. Addressing these issues will substantially strengthen rigor and reproducibility. See detailed comments below by sections.

**Response:**

**Conceptual coherence around multidimensional poverty**: Addressed by including a statement that discusses the need for a framework to capture the specific dimensions of multidimensional child poverty in African conflict settings.

**Consistency of inclusion criteria**: For the first objective, we will identify the dimensions of child poverty (during phase 1), and for the second objective, we will guide the identification of multidimensional aspects of child poverty with at least one dimension in the literature (during phase 2).

**Operationalization of armed conflict exposure:** The authors note that the armed conflict information is based on the study author’s descriptions of the conflict.

**Greater specificity regarding data extraction**: All concepts in this review are multilayered. Therefore, the data extraction would include specific information on children, armed conflict, countries in Africa, and dimensions of child poverty. For instance, the child concept will include all children (age-specific, gender specific, child-specific information such as street involvement, being an orphan child, HIV status, disabilities, unaccompanied minors, peacekeeper fathered, children conceived by rape (rape babies), children born of war (CBOW), and military children). Similar information related to armed conflict and the multidimensional aspect of poverty is attached to the supporting file S2_Table.

**Abstract**
1. In lines 29-31, the abstract states: “The articles should include at least two or more dimensions of child poverty in armed conflict countries in Africa.” However, in lines 149-151, the Concept section suggests inclusion of “any specific dimensions of child poverty.” These statements are inconsistent. The authors must clearly state whether eligibility requires (a) at least two dimensions, or (b) at least one dimension. For a scoping review, restricting inclusion to studies addressing two or more dimensions may unnecessarily exclude relevant literature.

**Response:** The objectives have been corrected both in the abstract and manuscript to identify underrepresented multidimensional aspects of child poverty (at least one or more) to include all relevant literature (lines 24 -26, 108-110,88-90).

2. There is no need to include the protocol registration link in the abstract section since it is in the main text. Similarly, lines 27 and 40-41 duplicate information about the protocol registration status.
**Response:** The authors have removed the protocol registration link in the abstract (line 46-48) and retained it in the manuscript. Also, the authors have removed duplicate information.

3. It would be important to briefly summarize the JBI protocol rather than just stating it as the case in line 39.

**Response:** The authors included a summary of the JBI protocol (lines 38-40) in the revised version.

4. There are several grammatical inconsistencies, such as “data bases” (line 33) and “will be search” (line 34). These should be corrected along with thorough proofread of the entire protocol to meet publication standards.

**Response:** The authors revised the abstract (objective, introduction, inclusion criteria, methods) proofread and corrected grammatical inconsistencies.

**Introduction**
5. The protocol describes health, education, and living standards as “global multidimensional indices”, then transitions into detailed descriptions of mortality, malnutrition, and education gaps (lines 78-95). However, it remains unclear whether the review conceptualizes these as: dimensions of poverty, consequences of conflict, or outcomes of poverty. The protocol would benefit from a clearer conceptual distinction between armed conflict exposure, multidimensional deprivation, and child outcomes.

**Response:** The reviewers conceptualized the inadequacies of the Global Multidimensional Poverty Index (MPI) and UNICEF’s MODA, clarifying the dimensions of armed conflict and stating that a framework will be developed at the end of this review (lines 81-82, 86-88).

6. Although the Global MPI and MODA are cited, the protocol does not explicitly state which framework will guide the categorization of poverty dimensions during analysis. Without a predefined structure, the mapping process risks becoming overly diffuse.

**Response**: A framework will be developed using Global MPI & MODA and conflict-specific dimensions of child poverty (lines 86-88).

7. The introduction section too long and also requires restructuring to ensure logical flow of information.

**Response**: The introduction is reduced from 90 lines (lines 52-103) to 51 lines.

**Review question and objectives**
8. Objective 2 states: “To map the missing dimensions of child poverty in armed conflict countries in Africa.” The phrase “missing dimensions” implies a predefined framework against which gaps will be assessed. If such a framework exists, it should be clearly specified earlier in the protocol. Otherwise, this objective should be rephrased to indicate identification of underrepresented domains in literature.

**Response:** The authors have restructured the objective 2 “to identify underrepresented multidimensional aspects of child poverty” against the Global MPI, UNICEF’S MODA and conflict-sensitive dimensions (lines 108-110).

**Inclusion and exclusion criteria**
9. Lines 149-151 state: “Studies will encompass any specific dimensions of child poverty, as well as articles that emphasize at least two or more dimensions of child poverty.” This wording creates confusion. The authors must clarify whether studies addressing only one dimension (e.g., nutrition, education, and WASH) will be included. This is a major methodological issue that requires resolution.
**Response**: The authors have clarified in the revised protocol, under the inclusion criteria, that the study will any dimensions of child poverty for objective 1 and one or more dimensions for objective 2 in the conflict region of Africa (lines 132-137).

10. The protocol defines children as those from birth to 18 years. However, it is unclear whether studies including mixed-age populations will be eligible if child-specific findings are reported. The authors should clarify this.
**Response:** The authors have clearly stated that the mixed age populations will be included when child- specific findings are reported (lines 117-118).

11. The protocol defines armed conflict as ≥25 battle-related deaths per year (UCDP definition). However, it does not specify how this criterion will be operationalized during screening. Will the authors independently verify conflict status using UCDP/ACLED, or rely on authors’ descriptions? Clear operational guidance is necessary to ensure reproducibility.

**Response:** The reviewers will rely on the author’s descriptions (line 142); however, if the literature reports any information on UNDP/ACLED, it will be captured in the S2 table of the data extraction tool (line 248). The author will share all data findings to ensure reproducibility in the final manuscript upon study completion (lines 261-262).

**Types of Evidence Sources**
12. Lines 161-164 include “knowledge synthesis articles (such as scoping reviews, systematic reviews, and narrative reviews).” Including review articles alongside primary studies raises concerns about duplication and conflation of evidence.
**Response:** The authors acknowledge this was an oversight and have removed the lines and the information concerning all the knowledge of synthesis articles**.**

**Methods and Search Strategy**
13. Main search terms should be stated in the main protocol, and the detailed search strategy should be kept as supplementary material. There is a lot of unnecessary detail in this section as well, e.g. meetings with librarian etc.

**Response:** The authors separated the searches from the main protocol and included them as supplementary material (S1 File) (lines 184). We have removed all the details of the process in this section.  
14. The protocol states that screening and extraction will be completed in 2026, with results expected in 2027. Given the scope of the review, this may be reasonable, but a brief justification of the timeline would improve clarity.

**Response:** The authors will screen the articles in 2026, map the dimensions in 2027, and have the results ready at the end of 2027, given the scope, volume, and complexity of the review as it interlocks with other parts of the research project (line 214-216).

**Data extraction**
15. The description about data in lines 248-251 is quite too general for reproducibility. The protocol should consider including in the extraction, parameters such as conflict type or duration, poverty measurement framework (MPI, MODA, DHS-based index, etc), specific dimensions assessed, age subgroup categories, and theoretical frameworks used by included studies. Including a draft data extraction table would significantly strengthen methodological transparency.

**Response:** The authors have attached a draft data extraction table (**S2 Table)** containing all parameters from the study articles, including all three concepts. Each concept is multilayered, and the details are attached for reproducibility (lines 221, 248).

**Data analysis and presentation**
16. Given the complexity of the topic, the authors should clarify how poverty dimensions will be categorized, whether qualitative findings will undergo thematic synthesis, whether findings will be stratified by region, conflict type, or age group, and how literature gaps will be identified and reported. Greater specificity is needed to demonstrate analytic rigor.

**Response**: The authors clarify that all poverty dimensions will be categorized using content analysis (line 246). All fine details of the population, children, Africa, and poverty are multilayered and will be captured in the draft data extraction tool (S2 table, lines 221-248) and the supporting material.

Reviewer #2: The Abstract should be structured to make it clear and ensure that all required content is included: Objective, Introduction, Inclusion criteria, and Methods.

**Response**: The abstract is revised and restructured as per the requirements, including objectives, introduction, inclusion criteria, and Methods (lines 24- 48).

Delete lines 41-44. Not necessary in the abstract. "We will use quality....

**Response:** The authors deleted all the unnecessary documents in the abstract.

Keywords: Reduce to a maximum of 5, arranged in alphabetical order

**Response**: The authors have arranged the 5 keywords in alphabetical order.

Data availability: As per the journal guidelines, since this is a protocol with no data but hope to collect data, the authors should state that "Deidentified research data will be made available when the study is completed and published."

**Response**: The authors included a statement that the research findings will be made available upon completion and publication of the study (lines 214–216).

Abbreviations: Write "JBI" in full the first time it is used.

**Response:** The authors have included the abbreviation in full, JBI with the Joanna Briggs Institute (JBI) in line 38.

Grammar: Typos and grammatical errors especially in the abstract. To be corrected.

**Response:** All typo and grammatical errors have been corrected in the abstract.

Review question and objectives: Should delete the two objectives and rewrite the review question to include the two objectives. The objective should be included in the introduction.

**Response:** Included the objectives in the introduction and the abstract. The objectives have been rewritten.

Context: The authors state, "This review will consider research papers from Africa." Please clarify whether it is papers from Africa or papers about Africa but may be written by authors outside of Africa. Or better still state that the study setting should be in Africa.

**Response**: Included the statement that the study setting should be in Africa (lines 144)

The manuscript has been read and approved by all authors. All three authors have contributed to preparing the manuscript, as per the International Committee of Medical Journal Editors (ICMJE) criteria for authorship, and no person other than the authors listed has significantly contributed to the manuscript. The content of this manuscript is our original work and has not been published in whole or in part. There is no conflict of interest.

The contents of the submission are included in the manuscript. The journal can choose reviewers who are passionate about global health and the thriving of children in Africa under a precarious conflict environment.

Thank you for your valuable time.

Sincerely,

Jasper Damaris Raja. Ph.D. Candidate, University of Saskatchewan, Canada [pxw184@mail.usask.ca](mailto:pxw184@mail.usask.ca)
