# Supplementary material for: Child poverty in armed conflict regions of Africa: A scoping review protocol
Source: PLoS One. 2026 Jun 29;21(6):e0352651. doi: 10.1371/journal.pone.0352651 (PMC13313329; doi:10.1371/journal.pone.0352651)
Supplement: Attachment — Submitted filename: Response_to_reviewers_auresp_2.docx [file pone.0352651.s007.docx]

Jasper Damaris Raja

107 Cumberland Avenue S

 Wollaston Hall, Saskatoon.

Saskatchewan, Canada SK S7N2R6

+13068801877

June 5, 2026

Dear Editor- in -Chief,

We received the reviewed manuscript titled “***Child Poverty in Armed Conflict Regions of Africa: A Scoping Review Protocol***” from PLOSOne. On behalf of myself and the other authors (Dr. Solina Richter and Dr. Kimberly Jarvis), I am **resubmitting the manuscript.** We have addressed **all points** raised by the **Academic Editor and the Reviewer.** Our responses are stated below with reference to the manuscript:

Reviewer 1

If applicable, we recommend that you deposit your laboratory protocols in protocols.io to enhance the reproducibility of your results. Protocols.io assigns your protocol its own identifier (DOI) so that it can be cited independently in the future. For instructions see: [https://journals.plos.org/plosone/s/submission-guidelines#loc-laboratory-protocols](https://can01.safelinks.protection.outlook.com/?url=https%3A%2F%2Ftrack.editorialmanager.com%2FCL0%2Fhttps%3A%252F%252Fjournals.plos.org%252Fplosone%252Fs%252Fsubmission-guidelines%2523loc-laboratory-protocols%2F1%2F010f019de1fb66a6-00fe5cac-6bd7-4ac2-847d-cb627787484c-000000%2FQiEkHCpkk0JrmhSW6u8NmFzifiUgSYyA2_cBe_CU6FY%3D258&data=05%7C02%7Cpxw184%40mail.usask.ca%7Ca1a1bc81db77497fc12b08dea741646d%7C24ab6cd0487e47229bc3da9c4232776c%7C0%7C0%7C639132096485357068%7CUnknown%7CTWFpbGZsb3d8eyJFbXB0eU1hcGkiOnRydWUsIlYiOiIwLjAuMDAwMCIsIlAiOiJXaW4zMiIsIkFOIjoiTWFpbCIsIldUIjoyfQ%3D%3D%7C0%7C%7C%7C&sdata=RJj%2F276VGaGfGUz1BCDsBYDUv52Bmflo7W54ambj5HY%3D&reserved=0). Additionally, PLOS ONE offers an option for publishing peer-reviewed Lab Protocol articles, which describe protocols hosted on protocols.io. Read more information on sharing protocols at [https://plos.org/protocols?utm_medium=editorial-email&utm_source=authorletters&utm_campaign=protocols](https://can01.safelinks.protection.outlook.com/?url=https%3A%2F%2Ftrack.editorialmanager.com%2FCL0%2Fhttps%3A%252F%252Fplos.org%252Fprotocols%253Futm_medium%3Deditorial-email%2526utm_source%3Dauthorletters%2526utm_campaign%3Dprotocols%2F1%2F010f019de1fb66a6-00fe5cac-6bd7-4ac2-847d-cb627787484c-000000%2F0xfvkx2VTlVDZ3MtA4LAeCXGjE2M6qIP1x-fG9AL2Rk%3D258&data=05%7C02%7Cpxw184%40mail.usask.ca%7Ca1a1bc81db77497fc12b08dea741646d%7C24ab6cd0487e47229bc3da9c4232776c%7C0%7C0%7C639132096485395314%7CUnknown%7CTWFpbGZsb3d8eyJFbXB0eU1hcGkiOnRydWUsIlYiOiIwLjAuMDAwMCIsIlAiOiJXaW4zMiIsIkFOIjoiTWFpbCIsIldUIjoyfQ%3D%3D%7C0%7C%7C%7C&sdata=AFrSBbZu7RESvHnGysXt0x77%2Fa5IeIdt%2FGp8hvBsjhI%3D&reserved=0).

**Response**: Not applicable as it is not a laboratory protocol.

Please review your reference list to ensure that it is complete and correct. If you have cited papers that have been retracted, please include the rationale for doing so in the manuscript text, or remove these references and replace them with relevant current references. Any changes to the reference list should be mentioned in the rebuttal letter that accompanies your revised manuscript. If you need to cite a retracted article, indicate the article’s retracted status in the References list and also include a citation and full reference for the retraction notice.

**Response**:

The authors reviewed the reference list and adhered to PLOS one recommendation with minor changes in the published articles. We included two references (44 and 49) to strengthen the revised manuscript with relevant and current references (lines 121 & 126). Also, the reference list was cross-checked for retraction through a retraction watch database.

**Reviewer 2**

The authors have addressed all comments raised during the first round of review, which has substantially improved the manuscript’s quality and readability. Nevertheless, greater care is required to ensure that the revised manuscript is free of typographical errors. For example, lines 109–111 appear to be incomplete sentences.
**Response**: The authors have reviewed and completed the incomplete sentences and addressed the typo errors in 109-113.

Second, the Methods section requires reorganization. For example, the subsections “Inclusion and exclusion criteria” and “Types of evidence sources” should be placed within the Methods section. In addition, the subsections “Type of evidence sources” and “Source of evidence selection” are overlapping in scope and should be consolidated and shortened. Accordingly, the Methods section should be restructured to include the following subsections:
• Study Design
• Inclusion and exclusion criteria
• Types and sources of evidence
• Search strategy
• Data extraction
• Data analysis and presentation
• Ethics and dissemination

**Response:** The authors restructured and reorganized the method section from lines 118- 268 as per the suggested sub sections with

• Study Design
• Inclusion and exclusion criteria
• Types and sources of evidence
• Search strategy
• Data extraction
• Data analysis and presentation
• Ethics and dissemination

Also, the authors consolidated the overlapping sentences in the sections “Type of evidence sources” and “Source of evidence selection” from the lines 157-191.

The manuscript has been read and approved by all authors. All three authors have contributed to preparing the manuscript, as per the International Committee of Medical Journal Editors (ICMJE) criteria for authorship, and no person other than the authors listed has significantly contributed to the manuscript. The content of this manuscript is our original work and has not been published in whole or in part. There is no conflict of interest.

The contents of the submission are included in the manuscript. The journal can choose reviewers who are passionate about global health and the thriving of children in Africa under a precarious conflict environment.

Thank you for your valuable time.

Sincerely,

Jasper Damaris Raja. Ph.D. Candidate, University of Saskatchewan, Canada [pxw184@mail.usask.ca](mailto:pxw184@mail.usask.ca)
