# Supplementary material for: Child poverty in armed conflict regions of Africa: A scoping review protocol
Source: PLoS One. 2026 Jun 29;21(6):e0352651. doi: 10.1371/journal.pone.0352651 (PMC13313329; doi:10.1371/journal.pone.0352651)
Supplement: Peer review history

# Peer review: Decision Letter 0

**DOI:** 10.1371/journal.pone.0352651.r001

**Authors:** Olushayo Olu

18 Feb 2026

PONE-D-25-62077

Child Poverty in Armed Conflict Regions of Africa: A Scoping Review Protocol

PLOS One

Dear Dr. Raja,

Thank you for submitting your manuscript to PLOS ONE. After careful consideration, we feel that it has merit but does not fully meet PLOS ONE’s publication criteria as it currently stands. Therefore, we invite you to submit a revised version of the manuscript that addresses the points raised during the review process.

Please submit your revised manuscript by Apr 04 2026 11:59PM. If you will need more time than this to complete your revisions, please reply to this message or contact the journal office at plosone@plos.org. When you're ready to submit your revision, log on to https://www.editorialmanager.com/pone/ and select the 'Submissions Needing Revision' folder to locate your manuscript file.

Please include the following items when submitting your revised manuscript:

- A letter that responds to each point raised by the academic editor and reviewer(s). You should upload this letter as a separate file labeled 'Response to Reviewers'.
- A marked-up copy of your manuscript that highlights changes made to the original version. You should upload this as a separate file labeled 'Revised Manuscript with Track Changes'.
- An unmarked version of your revised paper without tracked changes. You should upload this as a separate file labeled 'Manuscript'.

If you would like to make changes to your financial disclosure, please include your updated statement in your cover letter. Guidelines for resubmitting your figure files are available below the reviewer comments at the end of this letter.

If applicable, we recommend that you deposit your laboratory protocols in protocols.io to enhance the reproducibility of your results. Protocols.io assigns your protocol its own identifier (DOI) so that it can be cited independently in the future. For instructions see: https://journals.plos.org/plosone/s/submission-guidelines#loc-laboratory-protocols. Additionally, PLOS ONE offers an option for publishing peer-reviewed Lab Protocol articles, which describe protocols hosted on protocols.io. Read more information on sharing protocols at https://plos.org/protocols?utm_medium=editorial-email&utm_source=authorletters&utm_campaign=protocols.

We look forward to receiving your revised manuscript.

Kind regards,

Olushayo Oluseun Olu

Academic Editor

PLOS One

Journal Requirements:

When submitting your revision, we need you to address these additional requirements.

1. Please ensure that your manuscript meets PLOS ONE's style requirements, including those for file naming. The PLOS ONE style templates can be found at

https://journals.plos.org/plosone/s/file?id=wjVg/PLOSOne_formatting_sample_main_body.pdf and

https://journals.plos.org/plosone/s/file?id=ba62/PLOSOne_formatting_sample_title_authors_affiliations.pdf

2. We notice that your supporting information (S1 Appendix) is included in the manuscript file. Please remove them and upload them with the file type 'Supporting Information'. Please ensure that each Supporting Information file has a legend listed in the manuscript after the references list.

3. If the reviewer comments include a recommendation to cite specific previously published works, please review and evaluate these publications to determine whether they are relevant and should be cited. There is no requirement to cite these works unless the editor has indicated otherwise.

[Note: HTML markup is below. Please do not edit.]

Reviewers' comments:

Reviewer's Responses to Questions

**Comments to the Author**

1. Does the manuscript provide a valid rationale for the proposed study, with clearly identified and justified research questions?

The research question outlined is expected to address a valid academic problem or topic and contribute to the base of knowledge in the field.

Reviewer #1: Yes

Reviewer #2: Yes

**********

2. Is the protocol technically sound and planned in a manner that will lead to a meaningful outcome and allow testing the stated hypotheses?

The manuscript should describe the methods in sufficient detail to prevent undisclosed flexibility in the experimental procedure or analysis pipeline, including sufficient outcome-neutral conditions (e.g. necessary controls, absence of floor or ceiling effects) to test the proposed hypotheses and a statistical power analysis where applicable. As there may be aspects of the methodology and analysis which can only be refined once the work is undertaken, authors should outline potential assumptions and explicitly describe what aspects of the proposed analyses, if any, are exploratory.

Reviewer #1: Yes

Reviewer #2: Yes

**********

3. Is the methodology feasible and described in sufficient detail to allow the work to be replicable?

Descriptions of methods and materials in the protocol should be reported in sufficient detail for another researcher to reproduce all experiments and analyses. The protocol should describe the appropriate controls, sample size calculations, and replication needed to ensure that the data are robust and reproducible.

Reviewer #1: Yes

Reviewer #2: Yes

**********

4. Have the authors described where all data underlying the findings will be made available when the study is complete?

The PLOS Data policy requires authors to make all data underlying the findings described in their manuscript fully available without restriction, with rare exception, at the time of publication. The data should be provided as part of the manuscript or its supporting information, or deposited to a public repository. For example, in addition to summary statistics, the data points behind means, medians and variance measures should be available. If there are restrictions on publicly sharing data—e.g. participant privacy or use of data from a third party—those must be specified.

Reviewer #1: Yes

Reviewer #2: No

**********

5. Is the manuscript presented in an intelligible fashion and written in standard English?

PLOS ONE does not copyedit accepted manuscripts, so the language in submitted articles must be clear, correct, and unambiguous. Any typographical or grammatical errors should be corrected at revision, so please note any specific errors here.

Reviewer #1: No

Reviewer #2: No

**********

6. Review Comments to the Author

Please use the space provided to explain your answers to the questions above and, if applicable, provide comments about issues authors must address before this protocol can be accepted for publication. You may also include additional comments for the author, including concerns about research or publication ethics.

You may also provide optional suggestions and comments to authors that they might find helpful in planning their study.

(Please upload your review as an attachment if it exceeds 20,000 characters)

Reviewer #1: Overall Assessment

This protocol addresses an important and underexamined area at the intersection of armed conflict, multidimensional poverty, and child health in Africa. The topic is timely, policy-relevant, and appropriate for the journal. The use of JBI methodology, OSF registration and dual screening procedures are clear strengths.

However, the protocol requires clarification in several core conceptual and methodological areas. The main concerns relate to; conceptual coherence around multidimensional poverty, consistency in inclusion criteria, operationalization of armed conflict exposure, and greater specificity regarding data extraction and synthesis. Addressing these issues will substantially strengthen rigor and reproducibility. See detailed comments below by sections.

Abstract

1. In lines 29-31, the abstract states: “The articles should include at least two or more dimensions of child poverty in armed conflict countries in Africa.” However, in lines 149-151, the Concept section suggests inclusion of “any specific dimensions of child poverty.” These statements are inconsistent. The authors must clearly state whether eligibility requires (a) at least two dimensions, or (b) at least one dimension. For a scoping review, restricting inclusion to studies addressing two or more dimensions may unnecessarily exclude relevant literature.

2. There is no need to include the protocol registration link at the abstract section since it is in the main text. Similarly, lines 27 and 40-41 is a duplication of information about the protocol registration status.

3. It would be important to briefly summarize the JBI protocol rather than just stating it as the case in line 39.

4. There are several grammatical inconsistencies, such as “data bases” (line 33) and “will be search” (line 34). These should be corrected along with thorough proofread of the entire protocol to meet publication standards.

Introduction

5. The protocol describes health, education, and living standards as “global multidimensional indices”, then transitions into detailed descriptions of mortality, malnutrition, and education gaps (lines 78-95). However, it remains unclear whether the review conceptualizes these as: dimensions of poverty, consequences of conflict, or outcomes of poverty. The protocol would benefit from a clearer conceptual distinction between armed conflict exposure, multidimensional deprivation, and child outcomes.

6. Although the Global MPI and MODA are cited, the protocol does not explicitly state which framework will guide categorization of poverty dimensions during analysis. Without a predefined structure, the mapping process risks becoming overly diffuse.

7. The introduction section too long and also requires restructuring to ensure logical flow of information.

Review Question and Objectives

8. Objective 2 states: “To map the missing dimensions of child poverty in armed conflict countries in Africa.” The phrase “missing dimensions” implies a predefined framework against which gaps will be assessed. If such a framework exists, it should be clearly specified earlier in the protocol. Otherwise, this objective should be rephrased to indicate identification of underrepresented domains in the literature.

Inclusion and Exclusion Criteria

9. Lines 149-151 state: “Studies will encompass any specific dimensions of child poverty, as well as articles that emphasize at least two or more dimensions of child poverty.” This wording creates confusion. The authors must clarify whether studies addressing only one dimension (e.g., nutrition, education, WASH) will be included. This is a major methodological issue that requires resolution.

Population clarification.

10. The protocol defines children as birth to 18 years. However, it is unclear whether studies including mixed-age populations will be eligible if child-specific findings are reported. The authors should clarify this.

11. The protocol defines armed conflict as ≥25 battle-related deaths per year (UCDP definition). However, it does not specify how this criterion will be operationalized during screening. Will the authors independently verify conflict status using UCDP/ACLED, or rely on authors’ descriptions? Clear operational guidance is necessary to ensure reproducibility.

Types of Evidence Sources

12. Lines 161-164 include “knowledge synthesis articles (such as scoping reviews, systematic reviews, and narrative reviews).” Including review articles alongside primary studies raises concerns about duplication and conflation of evidence.

Methods and Search Strategy

13. Main search terms should be stated in the main protocol and the detailed search strategy kept as supplementary material. There is a lot of unnecessary detail in this section as well e.g. meetings with librarian etc.

14. The protocol states that screening and extraction will be completed in 2026, with results expected in 2027. Given the scope of the review, this may be reasonable, but a brief justification of the timeline would improve clarity.

Data Extraction

15. The description about data in lines 248-251 is quite too general for reproducibility. The protocol should consider including in the extraction, parameters such as conflict type or duration, poverty measurement framework (MPI, MODA, DHS-based index, etc), specific dimensions assessed, age subgroup categories, theoretical frameworks used by included studies. Including a draft data extraction table would significantly strengthen methodological transparency.

Data Analysis and Presentation

16. Given the complexity of the topic, the authors should clarify how poverty dimensions will be categorized, whether qualitative findings will undergo thematic synthesis, whether findings will be stratified by region, conflict type, or age group, how literature gaps will be identified and reported. Greater specificity is needed to demonstrate analytic rigor.

Reviewer #2: The Abstract should be structured to make it clear and wnsure that all required content is included: Objective, Introduction, Inclusion criteria, and Methods.

Delete lines 41-44. Not necessary in the abstract. "We will use quality....

Key words: Reduce to a maximum of 5 arranged in alphabetical order

Dat availability: As per the journal guidelines, since this is a protocol wih no data but hope to collect data, the authors should state that " Deidentified research data will be made available when the study is completed and published"

Abbreviations: Write "JBI" in full the first time it is used.

Grammar: Typos and grammatical errors especially in the abstract.To be corrected.

Review question and objectives: Should delete the two objectives and rewrite the review question to include the two objectives. The objective should be included in the introduction.

Context: The authors states that " This review will consider research papers from Africa." Please clarify whether it is papers from Africa or papers about Africa but may be written by authors outside of Africa. Or better still sate that the study setting should be in Africa.

**********

7. PLOS authors have the option to publish the peer review history of their article (what does this mean?). If published, this will include your full peer review and any attached files.

If you choose “no”, your identity will remain anonymous but your review may still be made public.

**Do you want your identity to be public for this peer review?**  For information about this choice, including consent withdrawal, please see our Privacy Policy.

Reviewer #1: **Yes:** Robert Lubajo

Reviewer #2: **Yes:** Jayne Byakika Tusiime

**********

[NOTE: If reviewer comments were submitted as an attachment file, they will be attached to this email and accessible via the submission site. Please log into your account, locate the manuscript record, and check for the action link "View Attachments". If this link does not appear, there are no attachment files.]

To ensure your figures meet our technical requirements, please review our figure guidelines: https://journals.plos.org/plosone/s/figures

You may also use PLOS’s free figure tool, NAAS, to help you prepare publication quality figures: https://journals.plos.org/plosone/s/figures#loc-tools-for-figure-preparation.

NAAS will assess whether your figures meet our technical requirements by comparing each figure against our figure specifications.

# Author comment: Author response to Decision Letter 1

**DOI:** 10.1371/journal.pone.0352651.r002

6 Apr 2026

Thank you for your comments and reviews; they guided the authors in revising their manuscript.

Attachment Submitted filename: Response to Reviewers.docx

# Editor report: Decision Letter 1

**DOI:** 10.1371/journal.pone.0352651.r003

**Authors:** Olushayo Olu, Olushayo Olu

1 May 2026

PONE-D-25-62077R1

Child Poverty in Armed Conflict Regions of Africa: A Scoping Review Protocol

PLOS One

Dear Dr. Raja,

Thank you for submitting your manuscript to PLOS ONE. After careful consideration, we feel that it has merit but does not fully meet PLOS ONE’s publication criteria as it currently stands. Therefore, we invite you to submit a revised version of the manuscript that addresses the points raised during the review process.

Please submit your revised manuscript by Jun 15 2026 11:59PM. If you will need more time than this to complete your revisions, please reply to this message or contact the journal office at plosone@plos.org. When you're ready to submit your revision, log on to https://www.editorialmanager.com/pone/ and select the 'Submissions Needing Revision' folder to locate your manuscript file.

Please include the following items when submitting your revised manuscript:

- A letter that responds to each point raised by the academic editor and reviewer(s). You should upload this letter as a separate file labeled 'Response to Reviewers'.
- A marked-up copy of your manuscript that highlights changes made to the original version. You should upload this as a separate file labeled 'Revised Manuscript with Track Changes'.
- An unmarked version of your revised paper without tracked changes. You should upload this as a separate file labeled 'Manuscript'.

If you would like to make changes to your financial disclosure, please include your updated statement in your cover letter. Guidelines for resubmitting your figure files are available below the reviewer comments at the end of this letter.

If applicable, we recommend that you deposit your laboratory protocols in protocols.io to enhance the reproducibility of your results. Protocols.io assigns your protocol its own identifier (DOI) so that it can be cited independently in the future. For instructions see: https://journals.plos.org/plosone/s/submission-guidelines#loc-laboratory-protocols. Additionally, PLOS ONE offers an option for publishing peer-reviewed Lab Protocol articles, which describe protocols hosted on protocols.io. Read more information on sharing protocols at https://plos.org/protocols?utm_medium=editorial-email&utm_source=authorletters&utm_campaign=protocols.

As the corresponding author, your ORCID iD is verified in the submission system and will appear in the published article. PLOS supports the use of ORCID, and we encourage all coauthors to register for an ORCID iD and use it as well. Please encourage your coauthors to verify their ORCID iD within the submission system before final acceptance, as unverified ORCID iDs will not appear in the published article. *Only*  the individual author can complete the verification step; PLOS staff *cannot*  verify ORCID iDs on behalf of authors.

We look forward to receiving your revised manuscript.

Kind regards,

Olushayo Oluseun Olu

Academic Editor

PLOS One

Journal Requirements:

If the reviewer comments include a recommendation to cite specific previously published works, please review and evaluate these publications to determine whether they are relevant and should be cited. There is no requirement to cite these works unless the editor has indicated otherwise.

Please review your reference list to ensure that it is complete and correct. If you have cited papers that have been retracted, please include the rationale for doing so in the manuscript text, or remove these references and replace them with relevant current references. Any changes to the reference list should be mentioned in the rebuttal letter that accompanies your revised manuscript. If you need to cite a retracted article, indicate the article’s retracted status in the References list and also include a citation and full reference for the retraction notice.

Additional Editor Comments:

The authors have addressed all comments raised during the first round of review, which has substantially improved the manuscript’s quality and readability. Nevertheless, greater care is required to ensure that the revised manuscript is free of typographical errors. For example, lines 109–111 appear to be incomplete sentences.

Second, the Methods section requires reorganization. For example, the subsections “Inclusion and exclusion criteria” and “Types of evidence sources” should be placed within the Methods section. In addition, the subsections “Type of evidence sources” and “Source of evidence selection” are overlapping in scope and should be consolidated and shortened. Accordingly, the Methods section should be restructured to include the following subsections:

• Study Design

• Inclusion and exclusion criteria

• Types and sources of evidence

• Search strategy

• Data extraction

• Data analysis and presentation

• Ethics and dissemination

[Note: HTML markup is below. Please do not edit.]

**********

[NOTE: If reviewer comments were submitted as an attachment file, they will be attached to this email and accessible via the submission site. Please log into your account, locate the manuscript record, and check for the action link "View Attachments". If this link does not appear, there are no attachment files.]

To ensure your figures meet our technical requirements, please review our figure guidelines: https://journals.plos.org/plosone/s/figures

You may also use PLOS’s free figure tool, NAAS, to help you prepare publication quality figures: https://journals.plos.org/plosone/s/figures#loc-tools-for-figure-preparation.

NAAS will assess whether your figures meet our technical requirements by comparing each figure against our figure specifications.

# Author comment: Author response to Decision Letter 2

**DOI:** 10.1371/journal.pone.0352651.r004

5 Jun 2026

Jasper Damaris Raja

107 Cumberland Avenue S

Wollaston Hall, Saskatoon.

Saskatchewan, Canada SK S7N2R6

+13068801877

June 5, 2026

Dear Editor- in -Chief,

We received the reviewed manuscript titled “Child Poverty in Armed Conflict Regions of Africa: A Scoping Review Protocol” from PLOSOne. On behalf of myself and the other authors (Dr. Solina Richter and Dr. Kimberly Jarvis), I am resubmitting the manuscript. We have addressed all points raised by the Academic Editor and the Reviewer. Our responses are stated below with reference to the manuscript:

Reviewer 1

If applicable, we recommend that you deposit your laboratory protocols in protocols.io to enhance the reproducibility of your results. Protocols.io assigns your protocol its own identifier (DOI) so that it can be cited independently in the future. For instructions see: https://journals.plos.org/plosone/s/submission-guidelines#loc-laboratory-protocols. Additionally, PLOS ONE offers an option for publishing peer-reviewed Lab Protocol articles, which describe protocols hosted on protocols.io. Read more information on sharing protocols at https://plos.org/protocols?utm_medium=editorial-email&utm_source=authorletters&utm_campaign=protocols.

Response: Not applicable as it is not a laboratory protocol.

Please review your reference list to ensure that it is complete and correct. If you have cited papers that have been retracted, please include the rationale for doing so in the manuscript text, or remove these references and replace them with relevant current references. Any changes to the reference list should be mentioned in the rebuttal letter that accompanies your revised manuscript. If you need to cite a retracted article, indicate the article’s retracted status in the References list and also include a citation and full reference for the retraction notice.

Response:

The authors reviewed the reference list and adhered to PLOS one recommendation with minor changes in the published articles. We included two references (44 and 49) to strengthen the revised manuscript with relevant and current references (lines 121 & 126). Also, the reference list was cross-checked for retraction through a retraction watch database.

Reviewer 2

The authors have addressed all comments raised during the first round of review, which has substantially improved the manuscript’s quality and readability. Nevertheless, greater care is required to ensure that the revised manuscript is free of typographical errors. For example, lines 109–111 appear to be incomplete sentences.

Response: The authors have reviewed and completed the incomplete sentences and addressed the typo errors in 109-113.

Second, the Methods section requires reorganization. For example, the subsections “Inclusion and exclusion criteria” and “Types of evidence sources” should be placed within the Methods section. In addition, the subsections “Type of evidence sources” and “Source of evidence selection” are overlapping in scope and should be consolidated and shortened. Accordingly, the Methods section should be restructured to include the following subsections:

• Study Design

• Inclusion and exclusion criteria

• Types and sources of evidence

• Search strategy

• Data extraction

• Data analysis and presentation

• Ethics and dissemination

Response: The authors restructured and reorganized the method section from lines 118- 268 as per the suggested sub sections with

• Study Design

• Inclusion and exclusion criteria

• Types and sources of evidence

• Search strategy

• Data extraction

• Data analysis and presentation

• Ethics and dissemination

Also, the authors consolidated the overlapping sentences in the sections “Type of evidence sources” and “Source of evidence selection” from the lines 157-191.

The manuscript has been read and approved by all authors. All three authors have contributed to preparing the manuscript, as per the International Committee of Medical Journal Editors (ICMJE) criteria for authorship, and no person other than the authors listed has significantly contributed to the manuscript. The content of this manuscript is our original work and has not been published in whole or in part. There is no conflict of interest.

The contents of the submission are included in the manuscript. The journal can choose reviewers who are passionate about global health and the thriving of children in Africa under a precarious conflict environment.

Thank you for your valuable time.

Sincerely,

Jasper Damaris Raja. Ph.D. Candidate, University of Saskatchewan, Canada pxw184@mail.usask.ca

Attachment Submitted filename: Response_to_reviewers_auresp_2.docx

# Editor report: Decision Letter 2

**DOI:** 10.1371/journal.pone.0352651.r005

**Authors:** Olushayo Olu, Olushayo Olu, Olushayo Olu

15 Jun 2026

Child Poverty in Armed Conflict Regions of Africa: A Scoping Review Protocol

PONE-D-25-62077R2

Dear Dr. Raja,

We’re pleased to inform you that your manuscript has been judged scientifically suitable for publication and will be formally accepted for publication once it meets all outstanding technical requirements.

Within one week, you’ll receive an e-mail detailing the required amendments. When these have been addressed, you’ll receive a formal acceptance letter and your manuscript will be scheduled for publication.

An invoice will be generated when your article is formally accepted. Please note, if your institution has a publishing partnership with PLOS and your article meets the relevant criteria, all or part of your publication costs will be covered. Please make sure your user information is up-to-date by logging into Editorial Manager at Editorial Manager® and clicking the ‘Update My Information' link at the top of the page. For questions related to billing, please contact billing support.

If your institution or institutions have a press office, please notify them about your upcoming paper to help maximize its impact. If they’ll be preparing press materials, please inform our press team as soon as possible -- no later than 48 hours after receiving the formal acceptance. Your manuscript will remain under strict press embargo until 2 pm Eastern Time on the date of publication. For more information, please contact onepress@plos.org.

Kind regards,

Olushayo Oluseun Olu

Academic Editor

PLOS One

Additional Editor Comments (optional):

Reviewers' comments:

# Editor report: Acceptance letter

**DOI:** 10.1371/journal.pone.0352651.r006

**Authors:** Olushayo Olu, Olushayo Olu, Olushayo Olu

PONE-D-25-62077R2

PLOS One

Dear Dr. Raja,

I'm pleased to inform you that your manuscript has been deemed suitable for publication in PLOS One. Congratulations! Your manuscript is now being handed over to our production team.

At this stage, our production department will prepare your paper for publication. This includes ensuring the following:

* All references, tables, and figures are properly cited

* All relevant supporting information is included in the manuscript submission,

* There are no issues that prevent the paper from being properly typeset

You will receive further instructions from the production team, including instructions on how to review your proof when it is ready. Please keep in mind that we are working through a large volume of accepted articles, so please give us a few days to review your paper and let you know the next and final steps.

Lastly, if your institution or institutions have a press office, please let them know about your upcoming paper now to help maximize its impact. If they'll be preparing press materials, please inform our press team within the next 48 hours. Your manuscript will remain under strict press embargo until 2 pm Eastern Time on the date of publication. For more information, please contact onepress@plos.org.

You will receive an invoice from PLOS for your publication fee after your manuscript has reached the completed accept phase. If you receive an email requesting payment before acceptance or for any other service, this may be a phishing scheme. Learn how to identify phishing emails and protect your accounts at https://explore.plos.org/phishing.

If we can help with anything else, please email us at customercare@plos.org.

Thank you for submitting your work to PLOS ONE and supporting open access.

Kind regards,

PLOS ONE Editorial Office Staff

on behalf of

Dr. Olushayo Oluseun Olu

Academic Editor

PLOS One
